# FDG-PET of Takayasu’s Arteritis

**DOI:** 10.1007/s11606-013-2695-7

**Published:** 2014-01-10

**Authors:** Kiyoshi Shikino, Takako Masuyama, Masatomi Ikusaka

**Affiliations:** Department of General Medicine, Chiba University Hospital, 1-8-1, Inohana, Chuo-ku, Chiba-city, Chiba Pref Japan

**Keywords:** Takayasu’s arteritis, PET/CT, aortitis

**Figure 1:**
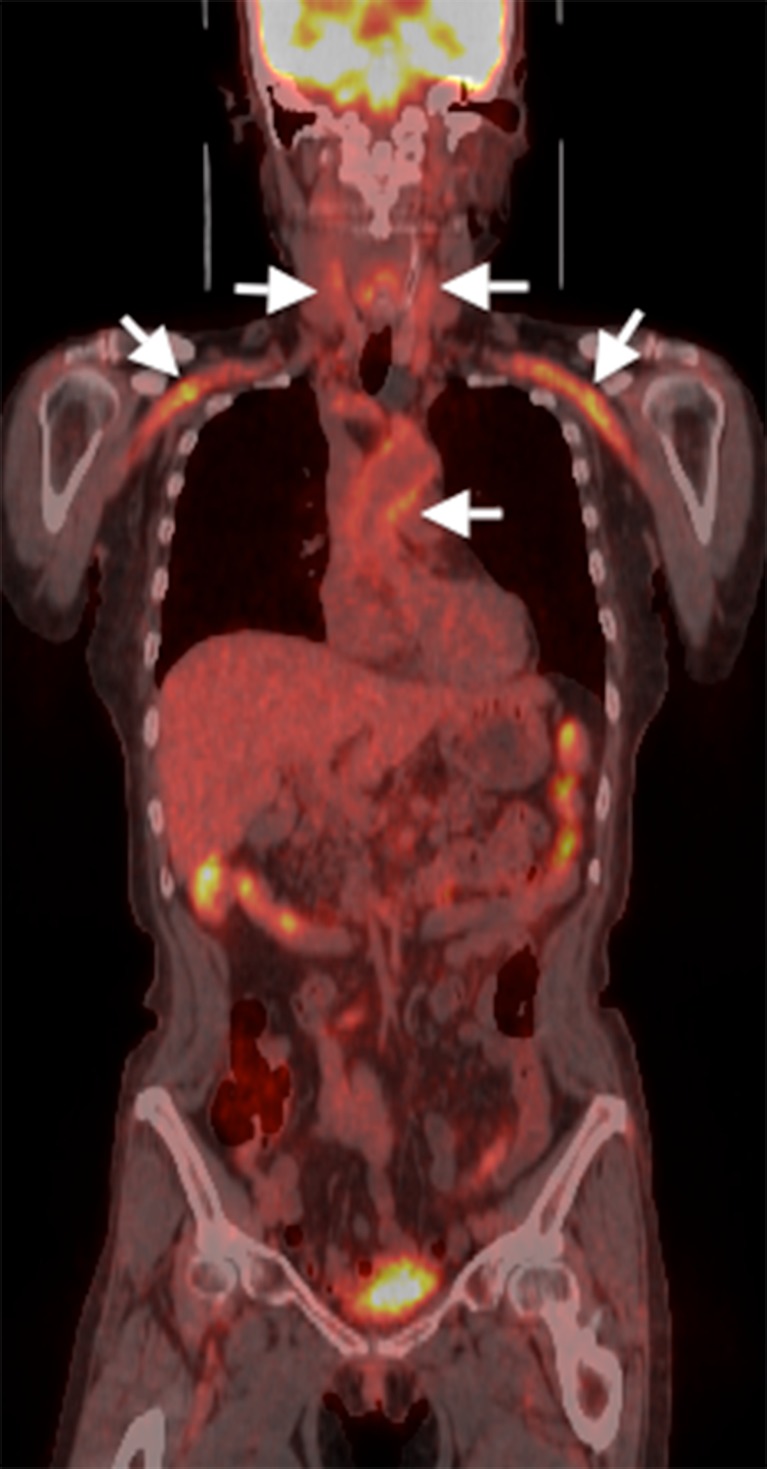
FDG-PET image with arrows pointing toward abnormal uptake in the carotid and subclavian arteries, as well as the thoracic aorta.

**Figure 2:**
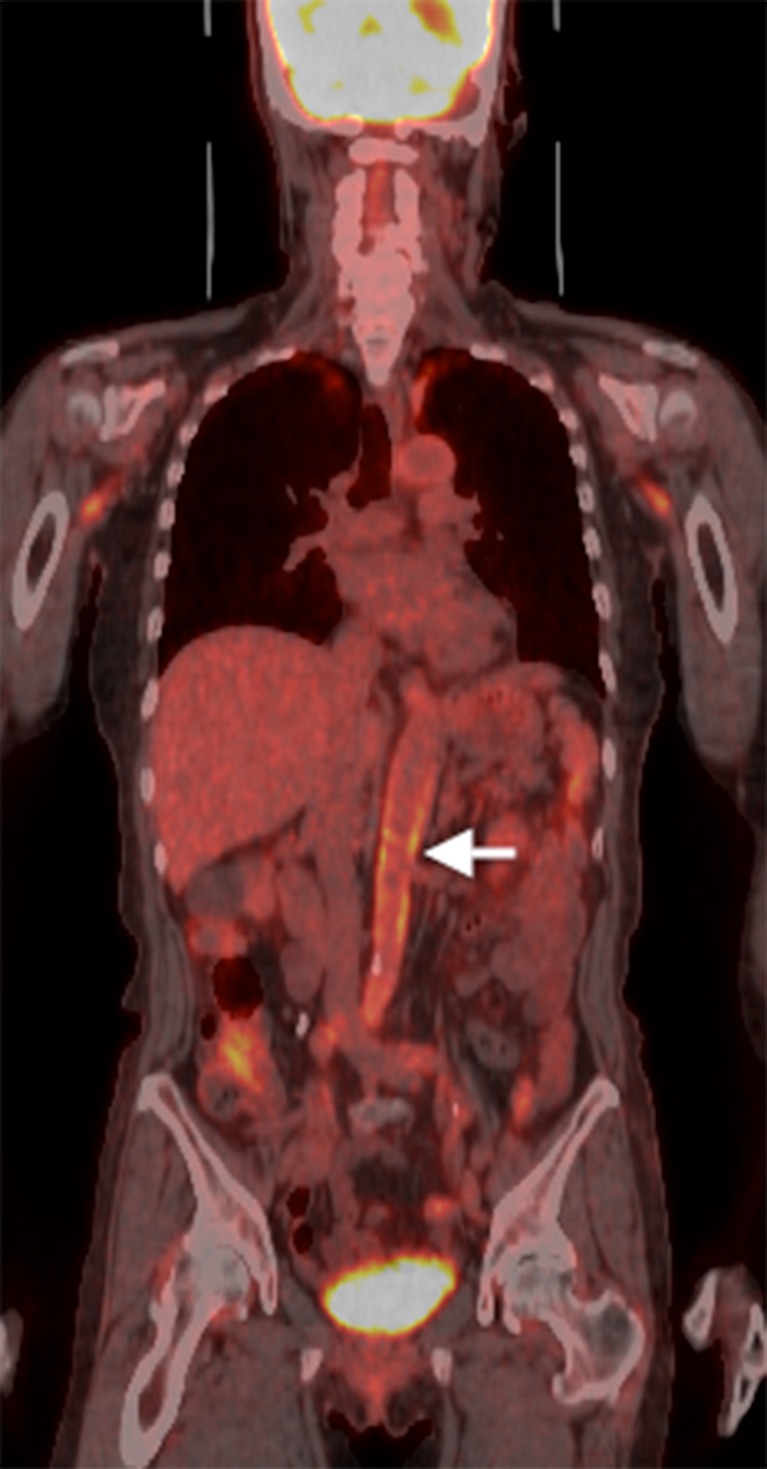
FDG-PET image with an arrow pointing to abnormal uptake in the abdominal aorta.

A 71-year-old woman presented with low-grade fever, appetite loss, and weight loss of four months' duration. She had fainted one month earlier during cervical extension and rotation. Physical examination revealed an abdominal aortic bruit and at least a 10 mmHg difference in systolic blood pressure between her arms. Fluorodeoxy glucose-positron emission tomography (FDG-PET) images showed increased FDG uptake in the thoracic aorta, abdominal aorta, as well as the carotid and subclavian arterial branches (Figs. 1 and 2). Takayasu’s arteritis was diagnosed. All symptoms improved with prednisolone 30 mg/day.

Takayasu’s arteritis is a chronic vasculitis of large vessels primarily affecting the aorta and its main branches. This disease is more common in young women, although age of onset varies.^1^ It has a worldwide distribution, with the highest prevalence in Asians. The early phase is characterized by nonspecific symptoms such as fever, malaise and weight loss. With disease progression, evidence of vascular insufficiency (including claudication of the arms) and neurologic symptoms manifest clinically. Syncope may result from subclavian steal syndrome or carotid sinus hypersensitivity.^2^ In this case, syncope while looking up and turning her head was caused by the latter. FDG-PET has a sensitivity of 92.6 % and a specificity of 91.7 % in active phase Takayasu’s arteritis.^3^
